
JOURNAL INFORMATION
==============================
NLM Title Abbreviation: ScientificWorldJournal
Journal ID: ScientificWorldJournal
NLM Journal ID: 101131163
Journal ID: TSWJ

The Scientific World Journal

ISSN: 2356-6140
EISSN: 1537-744X
Publisher: Wiley

ARTICLE INFORMATION
==============================
PMCID: PMC6084691
PMID: 30147625
DOI: 10.1100/tsw.2001.247
Article ID: 572390
Article version: 1
Subjects: Short Report

c-Abl, Free Radicals, and Osteoporosis

Li Baojie 1 *
Boast Sharon 1 3, *
Cong Feng 2
Ishiisect Tetsuro 4
Soh Jae-Won 1, *
Bouvier Nicole 1 3, *
Goff Stephen P. 1 3, *
Dept. of Biochemistry and Molecular Biophysics, Howard Hughes Medical Institute, Columbia University, College of Physicians and Surgeons, New York, NY 10032, USA
Dept. of Biology, Howard Hughes Medical Institute, Columbia University, College of Physicians and Surgeons, New York, NY 10032, USA
Howard Hughes Medical Institute, Columbia University, College of Physicians and Surgeons, New York, NY 10032, USA
Department of Biochemistry, Institute of Basic Medical Sciences, University of Tsukuba, Ibaraki, Japan
*Corresponding author.
Publication date: 2001
Electronic publication date: 2001 Dec 1
Volume: 1
First page: 148
Last page: 148
Copyright: Copyright © 2001 Baojie Li et al.
Copyright year: 2001
License: This is an open access article distributed under the Creative Commons Attribution License, which permits unrestricted use, distribution, and reproduction in any medium, provided the original work is properly cited.
License URL: https://creativecommons.org/licenses/by/3.0/

==============================
Miami *Nature Biotechnology* Short Reports

*TheScientificWorld* (2001) 1 (S3), 148SR

ISSN 1532-2246; DOI 10.1100/tsw.2001.247

### C-ABL, FREE RADICALS, AND OSTEOPOROSIS

Baojie Li*, Sharon Boast*‡, Feng Cong†, Tetsuro Ishii§, Jae-Won Soh*, Nicole Bouvier*‡, and Stephen P. Goff*‡

*Dept. of Biochemistry and Molecular Biophysics, †Dept. of Biology, ‡Howard Hughes Medical Institute, Columbia University, College of Physicians and Surgeons, New York, NY 10032; §Department of Biochemistry, Institute of Basic Medical Sciences, University of Tsukuba, Ibaraki, Japan
* Corresponding authors.

**INTRODUCTION**. c-Abl is a non-receptor tyrosine kinase implicated in the response to growth factors and DNA damage[1]. Mice lacking c-Abl exhibit a variety of complex phenotypes, including a high perinatal death rate, a short lifespan[2,3], and osteoporosis due to defective maturation of osteoblasts[4]. Here we report that *c-abl-/-* osteoblasts are hypersensitive to free radicals from reactive oxygen species and nitric oxide (NO). Mutant osteoblasts showed increased induction of peroxiredoxin I (Prx I) in response to arsenate, a potent oxidative stress agent, and increased cell death upon treatment with arsenate, $H_2O_2$, cisplatin, nitroprusside, S-nitro-N-acetyl-penicillamine (SNAP), or DETA NONOate, in spite of a slight resistance to serum starvation as compared to wild-type controls.

**RESULTS.** The c-Abl kinase is activated by treatment with oxidants in addition to genotoxic stress, and is likely to participate in the response to oxidative stress. We tested whether induction of Prx I was affected by the absence of c-Abl by using primary calvarial osteoblasts isolated from day 20 *c-abl-/-* and control embryos. The osteoblasts were treated with different concentrations of sodium arsenate for 12 hours and expression of Prx I mRNA was assessed by Northern blot. We observed Prx I mRNA up-regulation at lower concentration of arsenate, and to higher maximal levels, in the *c-abl-/-* cells. Western blot analysis showed similar increases in Prx I protein in the mutant lines. Thus, the *c-abl-/-* cells are hypersensitive to arsenate, suggesting that c-Abl normally limits or negatively regulates Prx I induction.

To test whether the absence of c-Abl affects the rate of induction of cell death caused by oxidative stress, we treated *c-abl-/-* and control osteoblasts for 24 hours with different concentrations of arsenate and the percentage of dead cells was scored by trypan blue exclusion. Untreated cell populations, as well as cells treated with only 0.05 mM arsenate, contained less than 10% dead cells. Mutant cells treated with 0.1 mM arsenate were hypersensitive: whereas wild-type cultures showed less than 30% dead cells, *c-abl-/-* cultures showed more than 60% dead cells. At all concentrations, *c-abl-/-* osteoblasts consistently showed more cell death than control cells. Treatment with $H_2O_2$, and CDDP, a DNA damage drug that also generates free radicals, produced similar results (data not shown). These results suggest that *c-abl-/-* osteoblasts are more sensitive than the wild-type cells to several oxidative stresses.

Nitric oxide is generated in osteoblasts in response to pro-inflammatory cytokines, mechanical strain, and sex hormone. High concentrations of NO inhibits osteoblast growth and differentiation, and plays an important role in inflammation-induced osteoporosis. To test whether *c-abl-/-* osteoblasts show hypersensitivity to NO, three NO donor compounds were used to treat osteoblasts for 24 hours and the extent of cell death was determined. The mutant cells were significantly more sensitive to sodium nitroprusside, and also to SNAP and DETA NONOate (data not shown). These results indicate that *c-abl-/-* osteoblasts are hypersensitive to a variety of nitrosative stress.

In contrast to the killing by oxidative/nitrosative stress, serum starvation, which causes apoptosis in osteoblasts, induced slightly less cell death in the mutant osteoblasts than in controls, suggesting that the protective role of c-Abl is specific to oxidative/nitrosative stress. Furthermore, mouse embryonic fibroblasts (MEFs) isolated from *c-abl-/-* mice did not show hypersensitivity to arsenate, cisplatin, $H_2O_2$, nitroprusside, SNAP, or DETA NONOate (data not shown), indicating that the abnormal response is specific to osteoblasts. These results suggest that c-Abl may play very different roles depending upon the cell type.

**DISCUSSION.** We propose that the hypersensitivity of *c-abl-/-* osteoblasts to oxidative and nitrosative stress contributes to the osteoporosis observed in mice lacking c-Abl. The hypersensitivity to free radicals and subsequent accumulation of oxidation and/or nitrosylation damage may contribute to the defects in maturation of *c-abl-/-* osteoblasts. Further, the number of osteoblasts would be reduced in the mutant mice due to their vulnerability to free radicals (oxidants and NO) generated in vivo. Our results suggest that c-Abl plays a special role in modulating the sensing of stress by osteoblasts. Phenotype of the *c-abl* knockout mice further indicates a correlation between osteoporosis and hypersensitivity of osteoblasts to free radicals.

**REFERENCES.**

1. Wang, J.Y. (2000) Regulation of cell death by the abl tyrosine kinase. Oncogene 19, 5643-5650
2. Schwartzberg, P.L. et al. (1991) Mice homozygous for the ablm1 mutation show poor viability and depletion of selected B and T cell populations. Cell 65, 1165-1175
3. Tybulewicz, V.L., Crawford, C.E., Jackson, P.K., Bronson, R.T., and Mulligan, R.C. (1991) Neonatal lethality and lymphopenia in mice with a homozygous disruption of the c-abl proto-oncogene. Cell 65, 1153-1163
4. Li, B. et al. (2000) Mice deficient in Abl are osteoporotic and have defects in osteoblast maturation. Nat. Genet. 24, 304-308
